# Development of an internationally agreed minimal dataset for juvenile dermatomyositis (JDM) for clinical and research use

**DOI:** 10.1186/s13063-015-0784-0

**Published:** 2015-06-12

**Authors:** Liza J. McCann, Jamie J. Kirkham, Lucy R. Wedderburn, Clarissa Pilkington, Adam M. Huber, Angelo Ravelli, Duncan Appelbe, Paula R. Williamson, Michael W. Beresford

**Affiliations:** Alder Hey Children’s NHS Foundation Trust, Eaton Road, Liverpool, UK; MRC North West Hub for Trials Methodology Research, Department of Biostatistics, University of Liverpool, Liverpool, UK; Infection, Immunology, and Rheumatology Section UCL Institute of Child Health, University College London, London, UK; Great Ormond Street Hospital NHS Foundation Trust, London, UK; Centre for Adolescent Rheumatology at University College London, University College London Hospital, London, UK; IWK Health Centre and Dalhousie University, 5850 University Avenue, Halifax, NS B3K 6R8 Canada; Università degli Studi di Genova and Istituto Giannina Gaslini, Via G. Gaslini 5, 16147 Genoa, Italy; Department of Women’s and Children’s Health, Institute of Translational Medicine, University of Liverpool, Liverpool, UK

**Keywords:** Juvenile, Childhood, Dermatomyositis, Idiopathic inflammatory myopathy, International, Collaboration, Dataset, Core outcome set, Core data set, Disease register, Clinical trials, Disease activity

## Abstract

**Background:**

Juvenile dermatomyositis (JDM) is a rare autoimmune inflammatory disorder associated with significant morbidity and mortality. International collaboration is necessary to better understand the pathogenesis of the disease, response to treatment and long-term outcome. To aid international collaboration, it is essential to have a core set of data that all researchers and clinicians collect in a standardised way for clinical purposes and for research. This should include demographic details, diagnostic data and measures of disease activity, investigations and treatment. Variables in existing clinical registries have been compared to produce a provisional data set for JDM. We now aim to develop this into a consensus-approved minimum core dataset, tested in a wider setting, with the objective of achieving international agreement.

**Methods/Design:**

A two-stage bespoke Delphi-process will engage the opinion of a large number of key stakeholders through Email distribution via established international paediatric rheumatology and myositis organisations. This, together with a formalised patient/parent participation process will help inform a consensus meeting of international experts that will utilise a nominal group technique (NGT). The resulting proposed minimal dataset will be tested for feasibility within existing database infrastructures. The developed minimal dataset will be sent to all internationally representative collaborators for final comment. The participants of the expert consensus group will be asked to draw together these comments, ratify and ‘sign off’ the final minimal dataset.

**Discussion:**

An internationally agreed minimal dataset has the potential to significantly enhance collaboration, allow effective communication between groups, provide a minimal standard of care and enable analysis of the largest possible number of JDM patients to provide a greater understanding of this disease. The final approved minimum core dataset could be rapidly incorporated into national and international collaborative efforts, including existing prospective databases, and be available for use in randomised controlled trials and for treatment/protocol comparisons in cohort studies.

## Background

It is recognised that an internationally agreed core dataset for use in routine clinical care could have numerous potential benefits for patients with this rare disease, with the additional advantage of informing future research [1]. The goals of this study are to reach international consensus on a minimal dataset (clinical, laboratory and patient/parent reported measures) for patients with juvenile dermatomyositis (JDM), which are practical, but cover the key variables that allow accurate assessment of disease activity and would measure change, such as response to treatment. Such a core set could then be rapidly incorporated into national and international collaborative efforts. To date, 2 distinct but overlapping core sets for JDM have been developed for use in clinical trials by the International Myositis and Clinical Studies (IMACS) group [2–4] and the Paediatric Rheumatology International Trials Organisation (PRINTO) [5–7]. These excellent tools have been principally designed for research and can be difficult to use in routine clinical practice due to time constraints. Our aim is to compliment these core sets with a separate internationally agreed minimal dataset for use in clinical practice. The key achievable objective of this study is for the developed minimal dataset to be recognised and accepted internationally to allow data collection over time on sufficient numbers of JDM patients. In order to achieve this, a formal consensus-driven methodology is required of all stakeholders.

Background work to this proposal is described elsewhere [1]. In brief, a working group of five JDM experts (CP, LW, AH, AR, LM) from the UK, Italy and Canada, representative of the major groups studying JDM and maintaining databases, scrutinised clinical and laboratory variables contained within current national and international collaborative databases. Variables contained within 2 or more databases were considered for inclusion in the provisional minimal dataset, informed by a literature search and detailed analysis of the UK Juvenile Dermatomyositis Cohort and Biomarker Study (JDCBS) [1, 8].

Details of this project have been included in the Core Outcome Measures in Effectiveness Trials (COMET) initiative database [9, 10]. In addition to close collaboration with COMET, our group has sought advice from senior members of the OMERACT (Outcome Measures in Rheumatology) collaboration, who are renowned for their work relating to outcome standardisation [11, 12].

## Methods/Design

A provisional dataset for JDM, developed through a formal process [1] has been used as a template to aid a structured multi-stage consensus process. The Delphi technique [13–15] has been used to gain the opinion of large groups of internationally representative clinicians caring for children and young people with JDM. This, together with a stepwise approach to gain the opinion of patients/parents, will help inform a detailed face-to-face nominal group consensus process by internationally representative JDM experts.

### Stage 1a – Delphi process to gain clinician opinion

A 2-stage survey technique (Delphi 1 and Delphi 2; with each stage building on the proceeding one) has been adopted, so that anonymous responses are obtained with equal influence to all individual participants [13–15]. An initial list of all potential variables was obtained from the provisional minimal database produced by this group [1] with outcomes listed individually but grouped under the relevant domain to aid interpretation by clinicians. Outcomes identified as patient/parent reported outcome measures or validated tools of muscle strength measurement [16] were presented within the appropriate domain as use of the tool rather than as individual components of the tool. However, in order to avoid discussions at this stage around which tool/instrument is better for measurement as opposed to another tool, an initial question asked participants how important they rate the use of a tool (without specifying which tool) to measure a certain domain, such as muscle strength or skin involvement. If they considered a tool to be important, they were asked which tool they favoured.

The list of variables was reviewed and approved by all authors as well as the research steering committee or leading representatives of each of the principal partner organisations (detailed in Table 1), including the International Myositis and Clinical Studies group – IMACS [4], Childhood Arthritis and Rheumatology Research Alliance – CARRA [17], Juvenile Dermatomyositis Research Group – JDRG (UK and Ireland), [18], Paediatric Rheumatology European Society – PReS JDM working party [19], and the Paediatric Rheumatology INternational Trials Organisation – PRINTO [7]. After modifications addressing feedback from these groups, the list (shown in Table 2) was formatted into a bespoke electronic questionnaire. The UK Paediatric Rheumatology Trainees’ Group and individuals from outside the UK selected by members of the JDM Minimal Dataset Steering Committee, piloted the questionnaire to determine average time taken to complete and readability of the questions.

**Table 1:** Details of collaborative groups and partner organisations

Collaborative organisation	Membership	Main geographical area covered	Collaborative role in study
Juvenile Dermatomyositis Research Group, JDRG (UK and Ireland) [18]	Multi-disciplinary healthcare professionals and scientists in the field of Paediatric Rheumatology; representative of all major UK paediatric rheumatology centres. JDRG members are all contributors to, and investigators in, the Juvenile Dermatomyositis Cohort and Biomarker Study, JDCBS [8]	UK and Ireland	▪ Delphi survey Email distribution
			▪ Representation in NGT meeting
			▪ Scrutinising data collection (Stage 3)
			▪ Aid dissemination of study results
			▪ Steering committee – study oversight
			▪ Patient/parent representation: steering committee and facilitated groups
Childhood Arthritis and Rheumatology Research Alliance, CARRA [17]	Paediatric rheumatologists and researchers throughout North America (n > 390); a proportion of whom have an interest in JDM and others more experienced in other areas of paediatric rheumatology	North America	▪ Delphi surveys Email distribution
			▪ Representation in NGT meeting and provisional minimal dataset
			▪ Aid dissemination of study results
Paediatric Rheumatology INternational Trials Organisation, PRINTO [7]	International research network with the aim of coordinating clinical trials and outcome studies in paediatric rheumatology	Initially a European collaboration but now includes > 50 countries and > 350 centres worldwide	▪ Delphi surveys via E-mail distribution
			▪ Representation in NGT meeting and provisional minimal dataset
			▪ Aid dissemination of study results
Paediatric Rheumatology European Society, PReS [19]	European scientific society for healthcare professionals in the field of paediatric rheumatology. The PReS JDM working group is a sub-group of this organisation that invites individuals with an interest or expertise in JDM	All European countries (EU and non-EU), extended to the Middle East. Associate members welcome worldwide	▪ Delphi surveys via Email distribution
			▪ Representation in NGT meeting
			▪ Aid dissemination of study results
International Myositis Assessment and Clinical Studies group, IMACS [4]	Coalition of healthcare providers and researchers with an interest in myositis syndromes. Myositis syndromes include JDM but also other inflammatory myopathies that are more common in adult patients	Part of the NIH science research programme, USA but welcomes members globally	▪ Delphi surveys via Email distribution
			▪ Representation in NGT meeting
			▪ Aid dissemination of study results
Euromyositis [39, 40]	European initiative leading to the creation of a web-based registry for adult myositis patients, recently expanded to include paediatrics	Initially European counties (EU and non-EU) expanded to include other collaborators outside Europe (Japan, Mexico, China, US)	▪ Collaborators in formation of a minimal dataset
			▪ Representation in NGT meeting
			▪ Testing data collection over time (Stage 3, potential)
OMERACT [11]	An independent initiative of international health professionals interested in outcome measures in rheumatology	North America, Europe and Asia-Pacific	▪ Advisory role in development of Delphi survey and in patient involvement
			▪ Representation in NGT meeting

*JDM*, juvenile dermatomyositis; *NGT*, nominal group technique; *NIH*, National Institute for Health

**Table 2:** Summary of questions asked in clinician survey compared to the parent and patient questionnaires

Domain	Questions asked of healthcare professionals in Delphi 1 and 2	Questions asked in parent questionnaire	Questions asked in young person’s questionnaire
General format of questionnaire	Two separate sections: Section A – when patient seen for their first visit only; Section B – all visits (first and subsequent). Asked to rate importance of each variable on a 1–9 scale separately for clinical use and for research	Asked to rate: How important do you think each one of these is when thinking about how well or unwell your child is due to JDM? (“not that important”/ “important”/or “really important”). Not asked separately about clinical/research	Asked to rate: How important do you think each one of these is when thinking about how well or unwell you are due to JDM (“not that important”/“important”/“really important”) Not asked separately about clinical/research
General questions on collecting and storing information and/or specimens	N/A	Asked to rate how important they think it is for all doctors and nurses/therapists to collect key information in the same way when they are looking after children with juvenile dermatomyositis (JDM)	Asked to rate how important they think it is for all doctors and nurses/therapists to collect key information in the same way when they are looking after children with JDM
	How important do you rate storing specimens at diagnosis for other biomarkers? (eg., DNA/serum/any other material)^a^	Asked to rate how important they think it is to store the type of information collected in clinic in a research database	Asked to rate how important they think it is to store the type of information collected in clinic in a research database
Demographics of patient	Date of birth	N/A	N/A
	Gender		
	Race		
	Ethnicity		
Family history	Of autoimmune disease	N/A	N/A
	Of neuromuscular disease		
Diagnostic data (general)	Age of onset of JDM	N/A	N/A
	Age of diagnosis of JDM		
MUSCLE – Diagnostic/activity data	Presence of symmetrical proximal muscle weakness: at diagnosis (section A) and every visit (section B)	Asking you if your child has any weakness	Asking if you have any weakness
	Presence of other muscle weakness (eg., distal or asymmetrical): section B only		
	Use of a validated tool to score muscle strength and/or function (options for different muscle strength scores)^a^	Testing to see how strong your child is	Testing to see how strong you are
SKIN – Diagnostic/activity data	Heliotrope rash (sections A and B)	Asking you if your child has any skin rashes	Asking if you have any skin rashes
	Gottron’s papules/Gottron’s sign (sections A and B)		
	Nail-fold capillary changes (sections A and B)		
	Other characteristic JDM rash		
	Use of a validated skin tool for JDM (options given to choose specific tools)^a^	Looking for rashes or skin signs that may suggest active JDM	Looking for rashes or skin signs that may suggest active JDM
SKIN – Additional activity data asked in section B only	Lipodystrophy	N/A	N/A
	Calcinosis		
	Cutaneous ulceration		
	Subcutaneous oedema		
	Malar/facial erythema		
	Shawl sign/V-sign		
	Mechanic’s hands		
	Alopecia		
	Vasculopathic lesions		
	Photosensitivity		
	Livido reticularis		
	Other erythema		
	Panniculitis		
MAJOR ORGAN INVOLVEMENT due to myositis – asked in section B only of clinician survey, plus patient/parent questionnaires	Musculoskeletal involvement (specific questions added for arthritis/contractures)^a^	Asking if your child has any muscle or joint pains	Asking if you have any muscle or joint pains
		Looking for swelling in any of the joints	Looking for swelling in any of the joints
	Gastrointestinal involvement (specific questions added for dysphagia/abdominal pain or GI ulceration)^a^	Asking if your child has any difficulty with swallowing/eating or if they have tummy pain	Asking if you have any difficulty with eating or if you have tummy pain
	Pulmonary involvement suggesting interstitial lung disease	Asking if your child has any shortness of breath or chest pain that may be related to JDM	Asking if you have any shortness of breath or chest pain that may be related to JDM
	Cardiac involvement		
CONSTITUTIONAL SYMPTOMS due to myositis – asked in section B only of clinician survey and in patient/parent questionnaire	Fever (>38 °C) due to myositis	N/A	N/A
	Weight loss (>5 %) due to myositis	N/A	N/A
	Fatigue due to myositis	Asking if your child feels tired due to JDM	Asking if you feel tired due to JDM
		Asking how tired your child feels using a formal “fatigue scale”	Asking how tired you feel using a formal ‘fatigue scale’
	Irritability due to myositis	Asking if your child feels irritable or miserable due to JDM	Asking if you feel irritable or miserable due to JDM
	Raynaud’s phenomenon	Asking if your child gets colour changes in their hands in cold weather	Asking if you get colour changes in your hands in cold weather
Growth	Height of patient (section B only)	Checking how well your child is growing (height)	Checking how well you are growing
	Weight of patient (section B only)	Asking about any weight loss and checking your child’s weight	Asking about any weight loss and checking your weight
Development/puberty	N/A	Asking about how your child is developing or how puberty is progressing	Asking about how you are developing or how puberty is progressing
Other information	Presence of malignancy (section B only)	N/A	N/A
	Patient having ongoing follow-up at this centre? (If no: options given for reason why)^a^		
Global disease activity	Use of a physician-scored measure of global disease activity - such as physician Visual Analogue Scale; VAS (specific choices for activity scores given)^a^	Asking your doctor to mark on a 0–10 cm scale how well or unwell they think your child has been based on what you tell them and what they see at your clinic visit	Asking your doctor to mark on a 0–10 cm scale how well or unwell they think you have been
Patient/parent Reported Outcome Measures (PROMs), Quality of Life/School issues	Use of a patient/parent reported outcome measure of disease activity or use of a validated measure of function (such as patient VAS/CHAQ) and/or a tool to measure Quality of Life (QoL) (specific choices given)^a^	Asking you or your child to complete a questionnaire that looks at how easy or difficult it is for them to do things like get dressed, have a bath, do activities	Asking you or to complete a list of questions that look at how easy or difficult it is for you to do things like get dressed, have a bath, do activities
		Asking you/your child to mark on a 0–10 cm scale how well or unwell your child has been over the last 4 weeks	Asking you to mark on a 0–10 cm scale how well or unwell you have been over the last 4 weeks due to JDM
		Asking you/your child to mark on a 0–-10 cm scale how much pain your child has had in the last 4 weeks due to JDM	Asking you to mark on a 0–10 cm scale how much pain you have had in the last 4 weeks due to JDM
		Asking how many days your child has missed school or college due to JDM	Asking how many days you have missed school or college due to JDM
		Asking more questions about your child’s school – how are things? Are they able to keep up with peers?	Asking more questions about school – how are things? Are you able to keep up with your peers?
		Asking your child how they feel emotionally in relation to their JDM (questions relating to Quality of Life or mood)	Asking about your feelings in relation to your JDM
INVESTIGATIONS: diagnostic data/disease activity data	Elevation of muscle enzymes at diagnosis (section A) or later in disease course (section B). (Questions asked about which specific enzymes to measure)^a^	Taking blood tests to monitor how active the disease is	Taking blood tests to monitor how active the disease is
	Electromyography (EMG) changes of myositis at diagnosis (section A)		
	Muscle biopsy evidence of myositis at diagnosis (section A)		
	Magnetic Resonance Imaging (MRI) changes of myositis at diagnosis (section A)	Asking your child to have a scan such as an MRI scan of their muscles to monitor the disease	Asking you to have a scan such as an MRI scan of your muscles
	Section B only: abnormal investigations (imaging, histology or cardio-pulmonary function tests indicating flare of myositis). If “yes,” tick which investigations are abnormal		
	Anti-nuclear Antibody (ANA) positivity at diagnosis (section A only)		
	Myositis specific antibody (MSA) positive at diagnosis (section A only)		
	Myositis associated antibody (MAA) positivity at diagnosis (section A only)		
Treatment	Date treatment started (mm/yyyy)	Asking specific questions about your child’s medicines and how they make them feel? Any unwanted effects?	Asking specific questions about your medicines and how they make you feel? Any unwanted effects?
	Patient taking steroids? Yes/No (options for type of steroid given)^a^	Asking you about the medicines your child is taking at the moment	Asking you about the medicines you are taking at the moment
	Patient taking a Disease Modifying Anti-Rheumatic Drug (DMARD)? (Not including biologic DMARDS) Yes/No (with option to select name of drug from a list)		
	Patient taking a biologic? Yes/No (with option to select name of drug from a list)		
	Patient having physiotherapy and/or occupational therapy?	Asking you if your child is doing any physiotherapy exercises	Asking you if you are doing your physiotherapy exercises
Other questions	To determine practice and experience - primary role, patient group, number of paediatric/adult patients under their care, geographical region of practice and membership of societies/professional bodies	How would you prefer information to be asked of you? (eg., in clinic, questionnaire before clinic, questionnaire before you come to clinic)	How would you prefer information to be asked of you? (eg., in clinic, questionnaire before clinic, questionnaire before you come to clinic)
Further information	Are there any variables that you think are important for clinical care that are not currently included in the list above? If so, please state what these are (same question also asked separately for variables important for research)	Are there any other key questions that we have missed but that you think it is important for us to ask when you come to clinic, thinking about how well/unwell you/your child is? If so, what key questions are they?	Are there any other key questions that we have missed but that you think are important for us to ask when you come to clinic, thinking about how well or unwell you are? If so, what key questions are they?
		Is there anything in this list (or from your experience of bringing your child to clinic) that you do not think should be included or you think your child does not like? If so, what? Why do you think that these questions should not be included?	Is there anything in this list (or from your experience of coming to clinic) that you do not like? If so, what? Why do you think that these questions should not be included?

^a^Further detailed questions asked in hidden rows, exposed if the answer to the preceding question suggests that the participant thinks that the variable is important

N/A, not applicable

### Participant details

Participation for the Delphi surveys was invited via membership of IMACS, CARRA, JDRG, PReS JDM working party and PRINTO; representative of international paediatric rheumatology and myositis specialty groups. These organisations include clinicians (clinical academic and non-academic), scientists and allied health professionals with expertise in paediatric rheumatological conditions and juvenile-onset and adult-onset myositis (Table 1). The entire membership was contacted by Email and invited to participate in the two web-based questionnaires using a bespoke Delphi system. The only exception to this was the PRINTO membership. In view of the fact that PRINTO is a large and heterogeneous organisation with over 900 individual members, the PRINTO directors (approximately 400 members) were contacted. PRINTO directors represent all of the major paediatric rheumatology centres among member countries. Many Email recipients are part of more than one research organisation and in this case, they were asked to complete each survey round once only and identify which other groups they are members of (and, therefore, potentially part of more than one mailing).

### Delphi round 1

The study objectives were clearly stated including what was expected of each participant. Participants were reminded of the importance of completing the entire Delphi process and asked to complete each round within 6weeks of receipt of Email. Reminder Emails were sent on a fortnightly basis to aid completion of each round, with phrasing of reminders considered to maximise the response rates [20]. Participants who agreed to take part were asked to register, allowing a unique identifier to be allocated to enable tracking of attrition at each round and identification of the research group that the participant was responding from (IMACS, CARRA, PRINTO, PReS JDM working group or the UK JDRG). Contributors were asked to specify their predominant clinical role (clinician/scientist/allied health professional/trainee), their specialty (specialist or major interest in rheumatology/ neurology/dermatology/immunology/other), the age of patient that they predominantly care for (adult/paediatric) their experience in the specialty/condition (≥ or < 10 years), and their region/country of practice. Participants were encouraged to answer the web-based survey to reflect their individual practice. However, in recognition that some respondents (particularly PRINTO directors) may answer on behalf of their centre, a question was asked to ascertain if they were answering as an individual reflecting their own practice, or on behalf of their department/centre (stating name of centre). Participants were not able to identify other participants or individual responses. Owing to their involvement in the study design and Delphi exercise, members of the JDM Minimal Dataset Steering Committee were not asked to participate in the Delphi surveys themselves, but encouraged maximal engagement and participation of individuals within the international research groups that they represented.

### Survey format

The outcome of this process will be to determine which variables clinicians perceive are important to include in one single minimal dataset that will be useful for clinical purposes and also inform research. However, within the survey, this question was divided into two separate sections, asking participants to rate in a structured format the importance of each variable for use in clinical practice, and separately, for its benefit in research. Each participant will be asked to score each of the outcomes listed using the Grade of Recommendations Assessment, Development and Evaluation (GRADE) scale of 1–9, with 1–3 labelled as “of low importance,” 4–6 labelled as “important but not critical for decision making” and 7–-9 labelled as “critical for decision making” [21]. Participants will have the option of selecting “unable to score” if they feel unable to comment based on their clinical experience, but they will be forced to answer each question before they move to the next stage of the questionnaire. For questions relating to investigations, in Delphi 1, participants will be asked whether each variable is accessible (easily available) to them in routine clinical practice in their hospital/country; given the option “yes,” “no” or “unable to comment”.

Participants who save an incomplete questionnaire will be contacted to encourage completion. Each participant will be given the opportunity to submit details of additional data items that they think should be included for clinical or research reasons (and reasons why) that are not part of the provisional data set. They will also be given the opportunity to submit written comments regarding those that have been included, including their opinion as to whether variables have been included that they perceive unnecessary, and if so, why.

### Analysis of round 1

Any additional outcomes suggested by participants in Delphi 1 will be carefully reviewed and coded by investigators to determine if they represent new outcomes not already incorporated in the questionnaire. Any identified will be added to the questionnaire for Delphi 2. For each outcome, the number of participants who have scored the outcome and the distribution of scores (as percentage who have scored each GRADE level) will be summarised. In view of the fact that the web-linked processes will involve participants with varying experience in JDM, any variables thought not to be useful will be noted at this stage but not specifically removed; respondents’ opinions will be recorded to help inform subsequent processes. The number of participants responding will be assessed following Delphi closure and results presented as total number of registrations, total number who completed the round and total number compared to potential respondents.

### Delphi round 2

Participants registering during part 1 will be invited to participate in the Delphi 2 survey and will thereby form the denominator for expected responses in round 2. In round 2, participants will be shown their score from round1 for each question (highlighted in every row of the Delphi 2) in addition to the total number of respondents for each question and the percentage of people responding to each ranking of importance (1–9) of the 9-point Likert scale. They will be asked to consider responses from the group and asked to verify that their round-1 response did indeed reflect their opinion, or given the opportunity to change or expand their response by re-scoring the outcome. Any changes to scores will be documented.

### Analysis of round 2

Round-1 responses will be compared between those who responded to both Delphi round1 and round2 against those who responded to Delphi round 1 only (but failed to respond to round2) to look for response bias. The total number of respondents to round2 will be recorded. For each outcome, the number of participants who have scored the outcome and the distribution of scores will be summarised. Content analysis will be used to code and describe written comments and responses. During the analysis of round2, results will be prepared for presentation at the consensus meeting within 3 groups: “consensus in,” “consensus out” or equivocal.

### Definition of consensus

1. “Consensus in” - consensus that the outcome should be included in the minimal dataset; defined as greater than 70 % of participants scoring 7–9; “critical for decision making”.
2. “Consensus out” - consensus that the outcome should not be included in the minimal dataset; defined as greater than 70 % of participants scoring as 1–3; “low importance”.
3. All other combinations will be presented as “equivocal”.

Seventy percent consensus has been chosen as a pre-defined cut off for this stage. The OMERACT handbook and COMET management group use this consensus definition.

### Sample size

There is currently no standard method for sample size calculation in Delphi processes and sample size estimates for this study have been carried out by a pragmatic approach considering responses from similar studies using a Delphi web-based survey distributed via rheumatological collaborations. Summating the current estimated membership of each collaborative working group (IMACS, CARRA, PRINTO, PReS JDM working party, JDRG) totals over 1000 members, but it is recognised that the majority of members will belong to more than one organisation and membership lists include retired or non-active members. Some organisations, such as IMACS, include many specialists in adult myositis who may feel less inclined to respond to a paediatric-focused study. Based on observed response rate from similar processes in paediatric rheumatology (49.8–84 %) [22–26] and attrition rate between 1.6 and 33 % [24–26], with less attrition in recent years, it is anticipated that the response rate for round1 will be approximately 150–200 participants with a 10 % attrition rate between rounds 1 and 2.

### Stage 1b – patient/parent engagement/consultation

A formal stepwise process for patient/parent participation will take place, distinct but alongside the Delphi process engaging clinicians and healthcare professionals, with both processes feeding into the nominal group consensus meeting of experts.

Currently, there is very little research published in JDM engaging patient/parent opinion. Our group has sought advice from senior members of COMET [9] and OMERACT [11] and consumer representatives of the National Institute for Health Research (NIHR) Clinical Research Network (CRN): Children/Arthritis Research UK Paediatric Rheumatology Clinical Studies Group (CSG) [27]. A sub-group of this organisation is the JDM Topic Specific Group (TSG), within which members of our group have been working to engage patients and parents in JDM research. Through this infrastructure, the opinions of young people with JDM and their parents/carers have been obtained, including checking and commenting on study information leaflets and patient/parent questionnaires. Our group is working directly with patient/parent groups within the UK and North America; Myositis UK [28] and Cure JM Foundation [29], who have agreed to distribute an electronic patient/parent questionnaire via their websites. In the UK, the research infrastructure within the JDRG allows patients and parents to be approached by the clinicians/healthcare professionals caring for them within their local centre [8, 18]. An attempt will be made by our collaborators to replicate the UK model in other counties to ensure international representation as far as is possible. Translation will take place as appropriate.

### Patient and Parent Questionnaire

The Delhi survey (sent to clinicians and healthcare professionals) has been reviewed by members of the JDM minimal dataset management committee and steering committee for relevance to patients and parents. There are many variables within this matrix that are not directly of interest to patients and parents (such as demographics) but others (such as patient/parent reported outcome measures) that are particularly relevant. Hence, a separate provisional questionnaire was designed for patients/parents to complete (Table 2), including some specific variables on school/quality of life felt to be important by young people when commenting on the questionnaires. A similar format for scoring responses to answers has been used as within the Delphi survey (GRADE scale) but simplified into three categories: “not that important,” “important,” or “really important”. Questionnaires were designed for screen-based modes of administration as well as paper format, depending on patient/parent preference. Electronic entry will be encouraged but patients/parents can choose to complete a paper questionnaire that will subsequently be entered into the electronic system by research staff.

Information leaflets were prepared for parents and for children/young people aged 8–11 years, 12–15 years, and 16+ years of age. Readability scores were used to check age appropriateness of the questionnaire and information leaflets [30, 31]. In addition, to ensure that the study documents were understandable to patients and parents, opinions were sought within the UK from the COMET [9] Patient and Public Involvement Coordinator, research nurses experienced in patient/parent involvement, consumer representatives of the Medicines for Children Research Network (MCRN) Clinical Studies Group [27], the Parent Group of the British Society for Paediatric and Adolescent Rheumatology (BSPAR) [32], young people participating in the NIHR CRN Young Person’s Advisory Group [33] and young people with JDM participating in a newly formed UK JDM Young Person’s Group, linked to the JDM TSG [27]. Subsequently, the final questionnaire, age-appropriate information leaflets, consent forms and assent forms were submitted to the NHS Integrated Research Application System and approved. The parent information leaflets make it clear that parents can either consent to be involved in the study themselves and/or consent for their child to participate in the study.

### Participants

Participation will be open to the following groups:

- Any child or young person with JDM
- Any adult who had JDM as a child
- Any parent of a child with JDM

There is no restriction in numbers and no exclusions within these groups. However, it is anticipated that children younger than 8 years of age are likely to be represented by their parents/carers and it is recognised that reliability or validity of child-reported measures at this age are often questionable [30]. Every effort will be made to translate information/questionnaires into the language of participants if needed or to engage translators. Patients with adult-onset myositis will not be allowed to participate, as there are significant differences in the disease processes and prognosis between adult-onset and juvenile-onset myositis. Adult-onset disease is defined in the UK as occurring after 16 years of age, and in the USA as occurring after 18 years of age.

### Distribution of questionnaires

Information about the study and questionnaires will be distributed in the UK as follows:

- Following site-specific ethics approval for this study at each major paediatric rheumatology centre already participating in the Juvenile Dermatomyositis Cohort and Biomarker Study (UK and Ireland) [8], parents and patients will be approached by their local doctors/nurses and asked whether they would be interested in taking part in this study. These doctors and nurses are part of the JDRG [18], including 14 major paediatric rheumatology centres in the UK, and are fully informed about the study and trained in Good Clinical Practice (GCP). After allowing time for consideration and the opportunity to ask questions, patients and or parents will be asked to complete a questionnaire when they come for their clinic appointment or at home.
- An advert will be submitted to patient/parent myositis organisations [28, 29] inviting participation. Patients and parents will be asked to complete a secure web-based questionnaire once they have confirmed that they have read the information leaflets and signed the consent form.
- Individuals who have previously expressed an interest in becoming involved in future research in JDM by contacting the UK Juvenile Dermatomyositis Research Group and/or by participation in parent/young peoples’ groups will be contacted by Email and invited to participate in this project.

Informed consent will be taken from each participant. Collaborators in Italy and the Netherlands will attempt to replicate this process (with country-specific ethics approval and translation as needed). Patients and parents in North America or elsewhere will be able to participate via electronic links provided by collaboration with patient and parent groups – Myositis UK [28] and Cure JM [29], with the recognised limitation that the questionnaires will only be provided in English-language format.

Currently, a number of patient and parent reported outcome measures are used in clinical practice and research in JDM [2, 3, 5, 6, 16, 34, 35]. Many of these tools have been developed for other rheumatological conditions (such as arthritis) rather than specifically for JDM. More recently, a JDM-specific tool – the Juvenile Dermatomyositis Multidimensional Assessment Report – has been developed and is currently being validated [36]. Young people with JDM participating in a newly formed UK JDM Young Person’s Group, linked to the JDM TSG [27], will be invited to participate in a focus group asking them to specifically consider various Patient Reported Outcome Measures currently available to determine if there is a preference for particular tools. Likewise, parents participating in a newly formed UK Parent Group, linked to the JDM TSG [27], will be asked to consider Parent Reported Outcome Measures. A detailed summary of the opinions of patients/parents will be prepared for participants of the consensus meeting and presented, with individual comments anonymised, to the group prior to and during the meeting.

All patient/parent participants completing the questionnaire will be asked if they would be willing to potentially participate in further stages of the study, and if so, asked to provide their contact details. Following analysis of the Delphi survey, they may be asked to consider specific questions or issues relating to the acceptability of completing variables in clinical practice, to help inform the consensus meeting, such as:

1. Time taken to complete each variable for the patient/parent.
2. Ways of completing variables that may make them more acceptable (for example, use of web-based questionnaires that can be complete before clinic).
3. Practical implications of completing each variable (for example, volume of blood needed for the test if extra blood tests indicated).
4. Consideration of validated patient/parent reported outcome measures that may form part of a minimal dataset to determine if there is a preference for particular patient/parent reported measures.

Consideration will be given to direct patient/parent involvement and representation during the nominal group consensus meeting, particularly if there are a few patient/parent representatives who stand out in being able to represent views of the group. If patients/parents are involved in the consensus meeting they will be fully supported throughout the process by the COMET Patient and Public Involvement Coordinator.

### Analysis of data

For each questionoutcome, the number (percentage) of participants who have scored the outcome as important and the distribution of scores will be summarised. Content analysis will be used to code and describe written comments with individual comments anonymised. A detailed summary of the opinions of patients/parents will be prepared for participants of the consensus meeting. Participants of the consensus meeting will be sent this information prior to the meeting and also be reminded of specific points relevant to variables during the meeting.

Other studies that members of our group are involved with are also engaging patient/parent participation that may be relevant to our study. For example, the European-wide FP7 funded SHARE (Single Hub and Access Point for Paediatric Rheumatology in Europe) project [37] led by Professor Nico Wulffraat, Utrecht, Netherlands, which aims to define a standard of care for paediatric rheumatology throughout Europe, includes a JDM work-stream. Thus, findings from this will also be summarized and be available for review by members of the nominal group consensus meeting.

### Stage 2 – Consensus meeting using the nominal group technique

The results of the Delphi exercise and patient/parent opinion will be used to inform Stage 2 of the study; a 2-day international consensus meeting of senior representative international experts in JDM tasked with undertaking nominal group consensus formation.

### Participants

The consensus meeting will include 15–20 healthcare professionals/scientists who are experts in JDM; many of whom are experienced in NGT. Patient/parent representation will be considered, as above. The total number of participants has come from recommendations for ideal NGT consensus group size [38] and suggestions from the COMET [9]/OMERACT [11] collaborative groups.

Individual experts have been chosen by the JDM steering committee with consideration of the following criteria:

1. Individual expertise: each individual is a recognised expert in the field of myositis.
2. Consideration of the wider groups that they lead or represent – ensuring that the final group includes representation from all international paediatric rheumatology and myositis expert groups.
3. Geographically internationally representative of the international paediatric rheumatology community.
4. Representative of the sub-specialty groups that care for children with JDM including paediatric rheumatology, neurology, dermatology and adult rheumatology.

### Structure of meeting

Analysis of the Delphi surveys will determine which variables were thought to be important to include in a minimal dataset by the wider group (“consensus in”) and which variables (“equivocal”) need more discussion within the expert consensus meeting. Summative feedback from the parent/patient questionnaires will be presented along with results of the Delphi (clinician opinion) for each variable to all participants of the consensus process prior to and during the meeting. Initially, members of the expert group will be presented with those variables judged as “consensus in” within the Delphi process to check for consensus agreement within the expert group before proceeding. It is anticipated that a quick decision can be made for a number of the variables in this way, but that certain items will need more detailed discussion to obtain consensus.

Nominal group technique (NGT) is a structured group meeting that follows a prescribed sequence of problem-solving steps [15], forcing equal participation among members in generating information and achieving outcomes. One non-voting participant, experienced in NGT but neutral to the study, will act as a facilitator and ensure that the process is not taken over by any one individual with strong views.

During the NGT process, the facilitator will frame the question to be discussed and show data from the Delphi surveys and patient/parent involvement relevant to that question. Each participant will have the opportunity to discuss his/her opinion for 1–2 minutes without interruption. Responses will be recorded and detailed notes taken. Participants will be given the opportunity to vote for their preferred response to each question. An agreement of ≥ 80 % consensus of all attendees will be required to consider each question as solved. If there is < 80 % consensus, participants will once again be given the opportunity to talk for 1–2 minutes. After discarding answers that are clearly not preferred, there will be another round of voting. The process will continue until there is consensus or lack of consensus achieved for each question. The NGT process will attempt to take into account feasibility of including individual variables (determined by results of the Delphi survey and patient/parent participation process) as well as the validity reliability of variables (from published literature) and the acceptability of the format of the dataset including definitions/glossary (defined in the provisional minimal dataset). Agreement with statements will be summarised by percentage agreement statistics.

### Stage 3 – Testing the dataset in real-life clinical practice

The proposed minimal dataset developed in Stage 2 will be tested for feasibility in Stage 3 of the study. This is to ensure that each outcome variable is feasible to complete in clinical practice and will directly inform clinical care as well as having a research rationale. Clinicians will be asked to enter anonymised data prospectively over time on at least 2 cases of JDM under their care via already-established internationally accessible secure web-databases [8, 39, 40] that will be adapted to incorporate questions asked within the developed minimal dataset. Each centre (hospital) has access to its own data with coded information from the complete database accessible with research steering committee approval. Each patient is assigned a unique identification number for research purposes and de-identified from personal information such as name, address, and hospital number. Data are protected by login and password and transfer of data encrypted. After allowing 6 months for data entry into collaborating databases, coded data on JDM patients will be made available for analysis. The study statistician will develop an algorithm to scan the dataset, flagging all missing observations, outlier and ill-coded variables to allow completion/data cleaning. In this way, Stage 3 will carefully assess whether the data entered into prospective data collections by clinicians (who are not necessarily experts in JDM) in real-world environments, is complete and accurate, as defined by the provisional dataset definitions/glossary [1] or whether clearer definition of variables are required. Definitions of variables will be refined by the JDM minimal dataset steering committee if needed, by Email or telephone consensus, after data analysis. The developed minimal dataset will be sent to the Chairs of all principal partner organisations’ (IMACS, CARRA, PReS, PRINTO, JDRG) individual research steering committees for final comment. The NGT group will be asked to draw together these comments and ratify and “sign off” the final minimal dataset.

### Ethical approval

Ethical approval has been obtained from the IPHS Research Ethics Committee (reference IPHS-1314-321). NHS Research Ethics Committee approval has also been obtained from the NRES Committee East Midlands (reference 14/EM/1259; IRAS project ID 160667).

### Ethical considerations

- Stage 1a (Delphi survey) does not involve patient data and simply involves consensus among clinicians
- Stage 1b (patient/parent involvement) will be canvasing opinion from patients (aged < 16 years of age) and their parents, as well as patients (>16 years of age) who have or previously had juvenile-onset myositis and parents of children with JDM. NHS Research Ethics approval obtained
- Stage 2 (nominal group consensus) does not involve patient data and simply involves consensus among experts in myositis
- Stage 3 will involve analysis of anonymised data after piloting the minimal dataset in a proportion of patients over a restricted period of time (6 months) via already established databases. Ethical approval for this stage is country specific, and although it will involve anonymised patient data, it is required for some collaborators. Ethical approval is already obtained for data collection within most of the organisations that may collaborate at this stage including the UK JDCBS, Euromyositis, the Gaslini Institute and CARRA. Other participants will be able to enter data via Euromyositis or obtain their own IRB approval. However, the study is not dependent on IRB approval of these individual countries, as sufficient patient numbers should be achieved via use of established databases that already include ethical approval. Our group will only have access, through research steering committee application, to secondary anonymised data collected within these individual databases.

## Discussion

The Delphi process has the advantage of allowing information to be exchanged between numerous individuals who may be geographically dispersed [38]. However, the advantage of face-to-face interaction is that it allows identification of the reasons for disagreements between individuals [38]. By using a combination of Delphi web-based surveys and face-to-face interaction in an NGT consensus group process, this study will obtain widespread opinion from a large number of clinicians in addition to the judgment of a small group of internationally renowned and representative group of experts in JDM. Patient and parent participation will be ensured via a structured process, including widespread distribution of a questionnaire and more detailed discussion within established patient/parent groups in the UK. The use of focus groups will allow stimulation of discussion and comparison of experiences across participants, whereas individual questionnaire responses will allow obtainment of opinions without influence from peers [30, 41]. In addition, consideration will be given to a small number of patient/parent representatives participating in the consensus meeting, representing the views of this group.

An internationally agreed minimal dataset has the potential to significantly enhance collaboration, allow effective communication between groups, provide a minimal standard of care and enable analysis of the largest possible number of JDM patients to provide a greater understanding of this disease. A consensus-driven, internationally approved minimum core dataset could be rapidly incorporated into national and international collaborative efforts, including existing prospective clinical databases, and be available for use in randomised controlled trials and for treatment/protocol comparisons in cohort studies.

### Study status

Currently at Stage 3: Delphi survey of clinicians and consensus meeting completed. Patient/parent questionnaires ongoing.

## Abbreviations

CRN: Clinical Research Network
CSG: Clinical Studies Group
BSPAR: British Society for Paediatric and Adolescent Rheumatology
CARRA: Childhood Arthritis and Rheumatology Research Alliance
COMET: Core Outcome Measures in Effectiveness Trials initiative
GCP: Good Clinical Practice
GRADE: Grade of Recommendations Assessment, Development and Evaluation
JDCBS: Juvenile Dermatomyositis Cohort Biomarker Study and Repository (UK and Ireland)
JDM: juvenile dermatomyositis
JDRG: Juvenile Dermatomyositis Research Group (UK and Ireland)
IMACS: International Myositis and Clinical Studies group
MCRN: Medicines for Children Research Network
NIHR: National Institute for Health Research
NGT: nominal group technique
OMERACT: Outcome Measures in Rheumatology
PReS: Paediatric Rheumatology European Society
PRINTO: Paediatric Rheumatology INternational Trials Organisation
SHARE: Single Hub and Access Point for Paediatric Rheumatology in Europe
TSG: Topic Specific Group
